# Perceptions of quality of life among Ugandan patients living with HIV: a qualitative study

**DOI:** 10.1186/1471-2458-14-343

**Published:** 2014-04-10

**Authors:** Doris Mutabazi-Mwesigire, Janet Seeley, Faith Martin, Achilles Katamba

**Affiliations:** 1 Makerere College of Health Sciences, Makerere University, P.O. Box 7062, Kampala, Uganda; 2 MRC/UVRI Uganda Research Unit on AIDS, P.O. Box 49, Entebbe, Uganda; 3 Department of Psychology, University of Bath, Bath, UK

**Keywords:** Quality of life, Resource-limited settings, Qualitative study, HIV/AIDS

## Abstract

**Background:**

Ugandans have endured the HIV epidemic for three decades. Now, with the availability of antiretroviral therapy (ART) and early diagnosis, those living with HIV can live longer and can enjoy the same life expectancy as the rest of the Ugandan population. This emerging trend necessitates the assessment of quality of life, alongside other patient outcomes, of those undergoing therapy, alongside other patient outcomes. While major strides have been made in developing measures of quality of life in the developed world, there remains a paucity of evidence from resource-limited settings. This challenge is further complicated by the contentious definition of quality of life, which is highly subjective and varies between individuals. In this paper, we aim to identify the determinants of quality of life for people living with HIV in a Ugandan context to contribute to the chronic care model for persons living with HIV/AIDS.

**Methods:**

Twenty HIV-positive participants took part in in-depth interviews at an urban clinic, with follow-ups at three and six months. Ten patients were on ART and ten not on ART. All interviews were transcribed and translated for analysis. Data were analysed manually using the framework approach to content analysis.

**Results:**

Individuals reported on four aspects of quality of life: liveability of the environment, utility of life, life ability of a person and appreciation of life. Respondents described multiple expectations and expressed hope for their future. However, many still suffered from stigma, fears of disclosure and poverty, which negatively affected their quality of life.

**Conclusions:**

Individuals living with HIV receiving treatment or in care experienced an improved quality of life in this setting, although the situation for many remains precarious.

## Background

Of the 34 million people living with HIV/AIDS globally, 23.5 million are in Sub-Saharan Africa [1]. Uganda alone has 1.1 million people living with HIV/AIDS out of a population of more than 30 million people [2]. According to figures reported in the Uganda AIDS Indicator Survey, the Ugandan government estimates HIV at 7.3% in 2012 compared with a prevalence of 6.4% seven years before [3,4]. This increase is partly due to a dramatic reduction in AIDS-related deaths coupled with increased access to antiretroviral therapy (ART). Between 2005 and 2011, the number of AIDS- related deaths in Sub-Saharan Africa reduced by 32% [1]. ART coverage has expanded markedly in Sub-Saharan Africa, with a reported 59% increase in the last two years. During this period, Uganda added an additional 100,000 people to its treatment programme [1].

With the increasing availability of ART and access to early HIV diagnosis, the life expectancy of HIV patients has improved. People living with HIV in Uganda now enjoy the same life expectancy as the general population [5]. With the transformation of HIV into a chronic condition through the immunological effectiveness of the treatment, health-related quality of life (HRQoL) has now become an important focus for HIV care [6].

Challenges surrounding lifelong treatment remain. Individuals living with HIV/AIDS in rural Uganda face a range of complex challenges in adapting to their condition [7]. Many people experience significant life disruption following diagnosis, which may include the loss of a spouse or child and must then make the transition to a “normal” life after initiating ART. However, some patients report difficulty in coping with living with HIV because of ongoing economic and social challenges.

While a number of studies have assessed HRQoL in Uganda, no qualitative study has addressed the topic in a prospective manner. One study of a home-based ART programme found improved scores in all domains of quality of life (QoL) such as role function, general health perceptions, and physical health [8]. At the start of the study low CD4 counts, high viral load, a lower level education, depression and socioeconomic dependence on other people were found to be predictors of poor HRQoL. At the end of the 12 months of follow-up, however, the only predictor for depressed QoL was economic dependence on others. Two other cross-sectional studies carried out in rural settings showed improved QoL in HIV patients participating in a community ART programme [9] and a positive association between both CD4 counts and informational support better and improved QoL functioning [10]. Another study in an urban setting in Uganda, reported better HRQoL among HIV patients on ART compared to those not receiving therapy [11].

HRQoL is a multi-dimensional concept and both its definition and assessment are contentious. However, it remains important to evaluate the HRQoL in an HIV-infected individual alongside the routine clinical outcomes. The World Health Organization (WHO) defines QoL as an “individual’s perception of their position in the context of culture and value systems in which they live and in relation to their goals, expectations, standards and concerns” [12]. QoL is highly subjective and unique to each individual, determined by experience, culture, beliefs, and personal values, and how these may relate to anticipated life outcomes [13]. Patients with significant health issues do not always present QoL scores corresponding to their physical status; subjective QoL may be higher than that predicted objectively from patients’ physical health. Only the individual can adequately assess his or her own personal QoL [14].

### Definitions and conceptualisations of QoL

Veenhoven [15] has proposed four aspects of QoL, which include “liveability of the environment, life ability of the individual, external utility of life and inner appreciation of life”. These facets of QoL provide the basis for a matrix comprising life chances, results and inner and outer qualities, which has been applied as a framework to evaluate common measures of QoL such as the Short Form (36) Health survey. An analysis using this matrix concluded that these measures typically only assess inner and not external QoL [15].

Another suggested approach to measurement of quality is the summation of “objective” and “subjective” components. Each of these components has seven domains; material wellbeing, health, productivity, intimacy, safety, place in the community and emotional wellbeing [16]. This approach does not fully address external QoL and may not adequately describe how the individual appreciates life or is generally satisfied with life [15]. These four qualities of life cannot be summed to give a single score of QoL, and QoL may be defined by how long and happily an individual lives [15]. Equating happiness with QoL also raises other challenges. Happiness and wellbeing have previously been defined in hedonic terms such as “presence of positive affect and absence of negative affect [17].”

In an effort to distinguish between wellbeing and happiness, other conceptions have drawn from a eudaemonic perspective and propose that wellness is not only about feelings but embraces the full conception of human functioning. Happiness then is a subjective feeling of positive versus negative affect [18]. This led to the development of the self-determination theory, which defines happiness in hedonic terms and wellness as a broader concept encompassing health and physical functioning [19]. Therefore, happiness may be an indicator, but not a guarantee, of wellness. One may be happy but not fully physically functional. The theory suggests that an individual thrives when they have autonomy, competence and relatedness [19]. Individuals with autonomy have a capacity for self-regulation, are able to use their abilities and are well-connected to the community and people around them. These represent basic psychological needs that give life satisfaction and determine QoL.

QoL has also been defined as satisfaction with life in general. An evaluation of different conceptual approaches to QoL can be explained with recourse to the following concepts: normal life, social utility, utility, happiness/affect, satisfaction with life, satisfaction with specific domains, achievement of personal goals and natural capacity. It has been suggested that defining QoL as satisfaction with life represents the best approach [20]. In addition to using life satisfaction or happiness as a measure of QoL, experiments on the factors mediating an individual’s degree of happiness or satisfaction have concluded that why and how one thinks about a past positive or negative event and one’s present affective mood together can determine happiness or life satisfaction [21].

Wellbeing has been closely associated with the attainment of needs and wants. One study from Thailand, for example, measured wellbeing using subjective methods [22]. Study participants identified health, food, family relationships, children, education, shelter, employment and overall satisfaction as key factors determining personal happiness.

The objective of the present study was to build on this understanding of QoL using subjective measures, and to elucidate the concept of QoL for people living with HIV in a Ugandan context to contribute to the development of a chronic care model for persons living with HIV/AIDS.

## Methods

### Study site and population

The study was carried out at an outpatient HIV clinic at the public national referral hospital, which serves people from a variety of cultural backgrounds. By the end of June 2013, the clinic had recruited a cohort of 19,089 patients, of whom 11,088 (58%) were on ART. Evaluations at this clinic focused on traditional clinical outcomes such as mortality, presence of opportunistic infections and progression of HIV. These patients had not been assessed for HRQoL prior to the current study.

### Study design

This qualitative study is a component of a larger ongoing prospective cohort study with a sample size of 1274 participants, of which 644 are receiving ART and 630 are receiving basic care that comprises of treatment for any opportunistic infection and daily co-trimoxazole tablets for prophylaxis. They are not eligible for ART. Each participant was followed up for a period of 6 months.

In-depth interviews (IDIs) were used to allow participants to share their experiences of adjusting to being HIV positive, undergoing ART (for those on ART) and discuss QoL issues stemming from their diagnosis. Between May and December 2012, 10 participants on ART and 10 not yet eligible for ART were selected. The age and gender distribution of these participants was similar to that of the clinical population, with an age range of 19–70 years and a gender distribution of 70–75% females. Data were collected through three IDIs: at baseline, and at 3 and 6 months’ follow-up. All the participants were interviewed by the first author. Interviews were not recorded using an audio device to encourage a free discussion of sensitive issues.

The initial interview aimed at creating rapport with participants and to get to know more about their life such as how long they have known their HIV status, reasons for HIV testing and whether they have children or spouses. Challenges they faced as persons living with HIV and what support mechanisms they could call upon were also discussed. The theme of QoL issues was introduced at this meeting and followed up in subsequent interviews, in which the interviewer probed for any changes in personal values and goals and how these related to QoL.

Participants were interviewed until theoretical saturation of the emerging themes was achieved. Themes were subsequently revisited and initial responses verified in follow-up interviews. At the end of each interview, the interviewer wrote a detailed transcript to ensure that all the discussions had been documented. These written scripts and the emerging concepts identified during the course of the interview were verified by the second author and any issues addressed. All interviews were transcribed and translated for coding and thematic analysis. Typed data were analysed manually using the framework approach to content analysis. We developed open codes, categories and themes using the raw data [23]. This was done by the first author and reviewed by the second author.

### Ethical considerations

Ethical approval to conduct the study was granted by the Research and Ethics Committee of Makerere University College of Health Sciences and the Uganda National Council for Science and Technology. Informed written consent was obtained from all study participants.

## Results

Of the 20 patients were enrolled in the study, 10 patients (two male) were eligible for ART and initiated treatment on the day of the first interview. The remainder (three male) were not eligible for ART, and received basic care. The age and sex distributions of the study participants are summarised in Table 1.

**Table 1:** Sample characteristics

**Variable**	**N**	**(%)**
Sex		
Male	5	25
Female	15	75
Age in years		
≤ 30	8	40
31–40	7	35
41–50	5	25

Gender and age distribution of the participants studied.

Of the 20 participants recruited, 18 completed three interviews. One woman on ART had one interview because she was transferred to a clinic near her home owing to difficulties in attending the clinic and one man receiving basic care completed only two interviews because he missed his second appointment and was subsequently seen four months after the first interview. Emerging themes were identified from the interviews, including “Live-ability” of the environment as indicators of QoL;“Life ability”, covering responses to treatment, stigma and stress; and Utility of life, specifically life expectations/goals and appreciation of life.

### Liveability of the environment

#### Indicators of quality of life

QoL was described in terms of general wellbeing and happiness influenced by good health, having money, good social relations and emotional wellbeing. The majority of the participants interviewed defined QoL in a manner very similar to the WHO definition of health as “a state of complete physical, mental and social wellbeing and not merely absence of disease or infirmity” [24].

One 45-year-old woman, receiving basic care, said that “QoL is about being happy, availability of food, shelter, clothes, and not being sick.” A 42-year-old woman remarked, “QoL is about the wellbeing of my children, their health and my health, and having shelter.” A 39-year-old man, receiving basic care said that “It is when one has all they wish to have. If I have enough money to take care of my family and I am able to work, my QoL is good.” Another man (23 years old) stated that “It’s about being healthy and able to work and earn money.”

The majority of the participants highlighted availability of money as the cornerstone of their wellbeing and hence a good QoL, for example “For me to enjoy life I need to have money” (female, 38 years) and “amidst poverty, nothing moves at all…” (female, 31 years). One 31-year-old man said “It’s all about having work to do and money and no worries as to where to get the next meal…as much as it’s important to be healthy physically and emotionally… we need food and other daily needs. It is impossible to be happy and peaceful when you have no money.”

Almost all interviewees mentioned that money and good health were important to their wellbeing and QoL. Male respondents were more candid when discussing money as a source of wellbeing compared with the women, who were a little more reticent about finances. For instance, one 31-year-old woman expressed QoL as “*egyeri yo yimiridemu*”, translated as “your standing in society”. When asked what exactly she meant by this, she explained that this referred to her financial situation.

Social relationships were also reported by some of the respondents as key to their wellbeing, for example, “Enjoying my life and the people around me and good relationships with them no quarrels.” (male, 41 years ), “It is important to be surrounded by people who love and care for you” (female, 48), and “a good relationship with my husband, my children’s wellbeing and good relationships with all my family members make me happy” (female, 33).

A lack of happiness due to poor social support was reported by one woman (aged 48). She had a 30-year-old son who she had left with the father when the son was still a baby. She had no close relationship with her son. She had also lost most of her siblings and her only living sister, who lived in the same village, did not wish to associate with her. She was nursing her mother (who was terminally ill with cancer and died just before the final interview). She said “nobody likes me; no one is available to help me… I have no joy.”

Emotional stability was a concern among a few of those interviewed. A 31-year-old woman described QoL as “Life with no worries, no stress…” As happiness was mentioned by a majority of the participants, we explored further what was meant by happiness in follow-up interviews. Happiness was seen as an indicator of QoL. The factors mentioned as sources of happiness included good family relations, good personal and family health, financial wellbeing and social outings. These findings were consistent across all ages and both genders.

One married 45-year-old woman explained: “I am happy when my children are healthy, have school fees and we have peace at home.” Another woman said “Good health and knowing that my family is healthy and well makes me happy—and of course when I have money” (27 years). A 23-year-old man said “My children and wife make me very happy and I enjoy their company.” A 50-year-old man in a polygamous marriage explained: “When my family and myself are healthy and we have all that we need I am very happy. When my family is happy, I am happy too.”

Two women interviewed related happiness and joy to their spiritual wellbeing (religion) and support. Both of them were Christians. “One of my happiest moments is when I am at church, praying and in fellowship with my church members,” said one married 45-year-old woman. The other woman (48 years, single) stated “the only time that I am happy is when I am in church praying and worshiping God.” Male respondents enjoyed going for a drink with friends, and watching football and plays. They derived joy and happiness from these activities: “I enjoy partying and taking alcohol with my friends” (male, 41), “I like talking and spending time with my friends” (male, 37) and “I love watching plays and football on TV, they make me happy” (male, 50). The female respondents’ sources of happiness were primarily related to family and social support, whereas male participants pointed to time out and having fun as sources of happiness.

Both men and women mentioned economic wellbeing as a major contributor to their happiness. According to one 29-year-old man, “… money is everything. If I had enough money, my life would be good.” A 37-year-old woman said “I am very happy when I am able to meet the financial needs of my children.” Finances were also mentioned as a source of unhappiness. “I do not think there is anything good about my life at the moment because my financial situation is so bad,” said one 31-year-old man who had lost his job. Similarly, a 31-year-old woman said “I feel terrible when I have no money; I am not a happy person because my finances are really bad. When we have little food, I have to give it up for my 3-year-old son and I stay without food. Sometimes I take a cup of black tea for my dinner.”

### Life ability

#### Response to care and ART

Participants that had been prepared to initiate ART at the baseline visit said that while they were ready to take the antiretroviral drugs (ARVs). Their main concern was of the potential side effects. Most had been somewhat unwell and were looking forward to a better health while on ART and would improve their wellbeing. A 23-year-old man, who had been unwell with an HIV-related infection, had been out of work for two weeks. He had been recently diagnosed with HIV and commented on starting ARVs “I am not anxious about taking ARVs and I believe they will make me feel better.” Similarly, one 30-year-old woman said “I have been really unwell for the last couple of months and I look forward to this treatment. I am positive it will make me feel better.” Finally, a 50-year-old man described his feelings, “I am happy to start the ARVs because my CD4 count had been declining…I pray they will not give me terrible side effects and my CD4 will begin to rise.”

A nursing assistant who had been unwell with fever and cough for more than a year and who was eager to start ART on her baseline visit reported that they would make her feel better compared with taking only co-trimoxazole as prophylaxis. A 30-year-old man who had been relatively well but had been instructed to start ART said “I am not anxious about ARVs. My prayer is that they will not give me terrible side effects and I remain as strong physically. I hope I will be able to continue with my routine work”. In contrast, some people described concerns, for example “I am worried about these drugs and how they will affect me. I hope I will not to get side effects such as vomiting, diarrhoea, change of skin/nail colour and rashes” (female, 30). Female participants expressed anxiety about possible side effects that may change physical appearance, such as weight gain and skin changes. Their body was very important to them.

At the second follow-up three months after the start of ART, most patients reported no side effects or only minor side effects. Those who had minor side effects said that they cleared within a few days and that they felt much better. Some perceived the side effects as very minor compared to the benefits of taking the ARVs: “Since I started taking ARVs, I have been fine” (female, 33). Another woman (23 years) said “The first few days may be three or four days were bad, I felt a lot of dizziness and later stabilised.” Similarly, a 25-year-old woman remarked “They did not affect me badly like I had anticipated. I had no side effects at all. There is great improvement in my health; I no longer get sickly with fevers and flu. I feel good and strong and I have gained weight.”

By the six-month visit, all the participants on ART reported a significant improvement and satisfaction with taking ARVs. All patients reported good adherence and had no problems taking the drugs because they had only one pill a day. A 25-year-old woman stated “I have not missed any dose and I have not had any problem with the drugs. I am able to continue with my daily work. The ARVs have helped a great deal.” Another woman (39 years) said “They have really helped me; I used to be unwell with fevers, had no appetite and felt tired all the time. I feel stronger; have no fevers and my skin and body feel and look better.” However, one 30-year-old woman revealed “Occasionally I vomit in the morning and I have asthma-like chest symptoms whenever the weather changes. However, these are so minor compared with how terrible I felt before the ARVs.” A 50-year-old man described benefits: “I feel as good and healthy as any other people and HIV is no longer a problem to me. I am so grateful for the availability of these drugs. My son and I are enjoying a normal life.”

However, despite these encouraging stories, the majority of participants who were not on ARVs did not want to start taking ARVs because they were concerned about side effects and the burden on the daily regimen. One respondent said “I am happy not taking ARVs and I pray that my CD4 count remains high. I fear having to take many pills.” A man (29 years) explained “I have a problem taking many pills, and what if I start ART and the free drugs run out… I hear they are very expensive on the open market.” In common with the female respondents that were on ART, those ones on basic care were concerned about side effects and any physical changes that may change their appearance or indicate that they have HIV. “I am worried about getting side effects such as rashes and my neighbours will know that I may be sick,” remarked a female participant not yet on ART. However, one woman receiving basic care wanted to start taking ART because she wanted to gain weight and look good like other patients she had seen at the clinic receiving ART. Both ART and basic care affected the physical and general wellbeing of all the participants.

#### Stigma

The majority of the women reported stigmatisation due to their HIV status and were worried about what family, friends and neighbours thought of them. Male respondents on the other hand seemed to have come to terms with their illness and did not care what other people said or thought of them. A 45-year-old woman said “I do not want my children to see me taking drugs. They know how to read and will understand that they are HIV drugs.” The same respondent reported that she did not like to come to this clinic every month as she feared that people who know her may find her at the HIV clinic. Similarly, on her second visit, a female participant aged 32 years said “I always pray when I am coming here that I do not meet anyone who knows me such as my friends or neighbours…. as I wait in the queue at the clinic, I am so worried. If it were possible I would cover my head and face.” A 42-year-old woman reported that she had stopped going to church when she developed a rash because she feared that other church members may discover her HIV status and speculate how she had been infected, despite her being a committed Christian. A 25-year-old woman on ART said that if other people were to discover her HIV status, they would judge her wrongly, assuming that she could be a prostitute despite the fact that it may take only one sexual encounter to acquire HIV. Many female respondents were concerned having HIV-related symptoms such as skin rashes and herpes zoster on exposed body parts, such as the face, that cannot be covered—thus making it clear to their community that they have HIV/AIDS. One 31-year-old woman remarked:

“I pray that I never get symptoms such Herpes zoster or skin rashes that may be an indication to my neighbours in the slum where I live that I am sick. I fear they will discriminate against me and my son and we shall be the major topic of discussion in the neighbourhood. I am positive that they would stop their children from playing with my son and no one will want to eat anything from my home and yet they many of them do not know their HIV status.”

Some of the women reported no sense of stigma on the first interview. However after establishing rapport, they reported feelings of shame stemming from their condition. The majority of males did not report any stigma at all in any of the three interviews. A 50-year-old man said “I do not mind if people know. I always share my experience and how good my life is with the HIV treatment. Many people do appreciate that I look and live a normal life like any of them so it does not bother me at all.” A 31-year-old man remarked “I don’t care if people know my HIV status. I have come to terms with it and I do not care what other people have to say.”

#### Stress/depression/anxiety/fear

The majority of the patients interviewed reported that they were fearful, shocked, and heartbroken and worried at the time of diagnosis. However, they received encouragement from family members, health workers and fellow patients in the clinic. Many were comforted when they started receiving care and found other people who had lived for many years following diagnosis and were doing well with or without ARVs. A 42-year-old woman described her reaction after her child tested HIV-positive: “I asked God why me, why my child? I was depressed.” Another 33-year-old woman, who was ready to begin ART, said that when she was diagnosed with HIV “I felt so low, I could not sleep, and I thought I was going to die.” The majority of respondents reported that the initial fear lasted only a couple of days or weeks and that they stabilised quickly once care was initiated. A 37-year-old woman receiving basic care said:

“My heart beat so fast, I was so scared and worried for my life; however, this did not last for long with the counselling, I got to know that the diagnosis was not a death sentence and I may live for a long time—and that there is the possibility to die from causes other than HIV/AIDS.”

A number of patients reported being worried about their children and how they would support them alongside coping with other social obligations to other family members. According to one 23-year-old man: “I mainly worry about my young children and if I died how they would survive…” A 37-year-old woman said “I definitely get worried because I may not live for long to support my children.” Low income was identified by all respondents as major cause of worry and anxiety. Some reported being unemployed, while others who were self-employed expressed concern that their businesses were not bringing in enough income. Participants expressed anxiety about house rent, school fees and food. “I have no money, and no job; these are my major problems,” remarked a 38-year-old woman on ART.

### Utility of life

#### Goals and expectations

All participants were hopeful and had set goals for their future life. There were no gender differences apart from the desire expressed by the majority of female respondents to gain financial independence from their male partners. All participants’ future plans included building their own homes, expanding their businesses, buying cars, ensuring the best education for their children, farming, securing a better job, having children and getting married for those that were unmarried. Plans were described:

“I would like to start a piggery project and expand my business” (female, 45), “I would like to build my own house as a woman; I would also love my husband to come to my parents’ home so that I officially introduce him to the family (traditional wedding) and to have a child with him” (female, 33), “I hope to make more money, build a shop for my business, and buy land and cows so that my wife and child are well catered for” (male, 23), “I hope to get married and have a child of my own” (male, 41) and “I plan to build my own home, send my child to a school of my choice and one day buy a car” (female, 30).

A 39-year-old single mother on ART said that she was planning to build more houses for her children so that even if she dies, they will have shelter and earn an income from the rentals. All participants reported that they appreciated life and hoped that they would live long enough to achieve their plans. Most participants did not change their plans over the six-month follow-up visits.

A few of the interviewees had changed habits such as drinking alcohol because of the illness, and put increased focus on planning for their families and children. For some, HIV had little impact on such behaviours: “My condition has not affected my life. I continue to work and my health has improved since I started treatment. I live my life the way I would have lived it without HIV,” remarked a 29 year old man. Conversely, a 38-year-old woman said “I stopped taking alcohol and having relationships with men because of my HIV status,” a 50-year-old man said the following:

“I used to take alcohol, but I have since stopped. I was renting small houses in the slums for my wives but ever since I got the diagnosis, I have built for them homes in my village and all my children that had dropped out of school are back in school. I became more focused after the diagnosis. I plan for the future of my wives and children.”

A 30-year-old woman reported “If I were not sick, I would have remarried and had other children.” The same respondent told the interviewer during the following visit that she plans to have another child after her business has stabilised. She said the father of her first child, who is unaware of her HIV status, is always asking her to have another child. Although she had tested HIV positive when she was expecting her first child, she was given preventive ART during pregnancy and her child is HIV-negative. She expressed hope that she could have another HIV-negative child. Related to this, a single mother on ART with one child said that after the diagnosis, she cut down her expenses on luxury items such as clothes and hair treatments. She explained she was saving every coin she makes to build a home for her child.

Many of the participants reported living a normal life with no dramatic changes after diagnosis with HIV and subsequent treatment. A female patient not on ART said “I sometimes forget that I have HIV…” A male patient also on ART also reported “I feel as good as other people I know….HIV is not my problem.” A 37-year-old man on his second visit reported “I do not worry about the illness because even people without HIV die.” However, almost all the participants still struggled with the fundamental challenges of living with HIV such as stigma, stress and fear of disclosure.

### Appreciation of life

QoL was commonly described as “*we yagala*”, translated as “being contented with oneself”. One 41-year-old male explained that “when one is contented with life (*we yagala*), they have no problems and whatever they set out to do comes out as desired.” A 39-year-old female said that “It’s when one is satisfied with themselves (*we yagala*), suffers no illness and has whatever they would like to eat, drink… and no worries.” Another female on ART said “It’s about feeling comfortable within your skin (*we yagala*), when you are healthy and have all your basic needs.” In addition, she used the expression “*Nga teli kubanja*”, meaning you are not indebted to anyone.

## Discussion

The results of this qualitative study are in accordance with the model of ‘four qualities of life’ [15]. Participants’ narratives alluded to the concept of “liveability of the environment”, which could be considered to describe the prevailing economic circumstances such as a lack of money, while the need for shelter could be said to pertain to “level of living”. Furthermore, “life ability of the person” could be defined as how capable one is able to cope with life problems. The participants in our study described feelings of stigma, resulting in non-disclosure of their HIV status. Women were most open about their feelings of shame attached to their condition. This may in turn undermine their ability to cope with chronic illness. However, these participants reported that they were in good health and living a normal life. The third theme that arose was “utility of life”, defined as life being “good for something more than itself”. The majority of patients remarked that their wellbeing was related to their perceived usefulness in society and factors such as being able to provide for their families and send their children to school. Finally, participants spoke of subjective wellbeing, expressed as “life in the eyes of the beholder” [15]. Many participants stated that they appreciate and enjoy life and good health. This was commonly described as being “contented with oneself”. Patients expressed hope for a long and good life without the threat of death or illness. Overall, QoL was perceived in terms of a long and happy life.

Happiness was commonly mentioned as an indicator of QoL by our study participants. Good social networks were themselves a source of happiness and hence good QoL, with the majority of the participants reporting that their families were a major source of happiness. Two female participants also mentioned the church and spiritual involvement as sources of joy. Our findings support those reported by a similar rural community in eastern Uganda, which has high rates of HIV and resulting comorbidities. In that study, participants in the process of rebuilding social networks and family structures acknowledged that involvement in church activities gave them a new life [25]. This connectedness has been proven to improve wellbeing when balanced with personal autonomy, as postulated by the self-determination theory [26].

Interestingly, while male participants presented happiness in purely hedonic terms as presence of positive affect [17], women described happiness in terms of relating to others and full physical functioning [18]. QoL was also described in terms of being comfortable within one’s own skin, which can itself lead to satisfaction with life. While this corresponds to a concept of overall QoL, it could also be considered a potential indicator of QoL [20].

As our participants described their QoL in detail, they spoke of the valuable things in their lives and how these related to their experiences of living with HIV. The ability to work and earn an income to sustain their daily needs and those of their families was described as a major component of QoL. Qualitative studies on wellbeing in developing countries, including Ethiopia, Thailand, Peru and Bangladesh, highlight the same themes and determinants of wellbeing found in the Ugandan study group. Food, shelter, money, health, family relations, children, education and work were commonly considered important for wellbeing in all population groups [27,28].

Access to ART has had a significant impact on QoL. When ART became available in developing countries more than decade ago, it not only gave hope to the patients but also resulted in positive changes in the livelihoods of people living with HIV. Many of the patients interviewed reported having experienced coming close to death, a loss of hope for the future, the loss of a partner or children and significant life disruption [7,25]. Similarly, after initiating ART, many respondents noted positive transformations such as overcoming stigma and social separation resulting from AIDS [29]. A study from South Africa elaborates in further detail on how patients regained health and social relations while in care [29]. Whereas study participants from a study in rural Uganda struggled to “get back to normal” and rebuild their social lives after a diagnosis of HIV and treatment [25], the present study, which took place in an urban setting almost ten years after availability of free ART, found that the majority of the patients were tested for HIV when they were still in relatively good health. Indeed, some even reported having initiated ART without any symptoms of HIV. Therefore limited life disruption was experienced by our participants thanks to the availability of support, counselling and the provision of ART. Participants have been able to live their lives as before, without disclosing their HIV status to most of their social contacts. Appreciation to the availability of treatment, most participants can look forward to a longer life, provide for their children and fulfil their life goals.

Despite this depiction of limited life disruption following diagnosis with HIV, participants’ narratives demonstrate that they continue to quietly suffer from stigmatisation, stress, poverty, uncertainty about the future and an inability to disclose their HIV status. Previous work has defined transition as when people reflect on their lives and take action [30]. It has been reported in other settings to be very important in determining the success of ART programs and to play an important role in managing chronic illness [31]. The narratives presented in the present study show that the participants have not reflected on their illness, which may in turn have long-term consequences on how they manage living with HIV.

This was confirmed by participants’ reported stigma and reluctance to disclose of their HIV status. Participants reported experiencing varying degrees of stigma. While one female participant said that if it were possible she would cover her face with a scarf at the clinic, another reported that if her neighbours discovered her status they may isolate her and her son and not allow their children to play with her son or eat in her home. These findings are similar to those of other studies in both the developed world [32] and other developing countries [27]. Owing to the perception of low social acceptance, few participants reported disclosure of their HIV status to their community. Challenges reported by non-disclosing HIV patients included stress, lack of support and of the threat of stigmatisation [33]. Interestingly, a qualitative study in five African countries including Tanzania identified disclosure as one coping mechanism for dealing with stigma [34]. However, Ugandan women who had been on ART treatment for a year also reported a lack of social support and the threat of stigma or discrimination as concerns [35]. These challenges surrounding social stigma and non-disclosure of HIV status negatively affected respondents’ QoL and still persisted even after one year of therapy.

### Limitations

All interviews were carried out by the first author and this may have caused some bias however transcripts were reviewed by the second author. Interviews were not tape recorded because some of the participants were not comfortable with the practice due to stigma and non disclosure of the status. The participants freely talked about their experiences and sometimes asked the interviewer not to write some of the points they reported.

## Conclusions

The present study captures the multidimensional nature of QoL and provides a holistic picture of the challenges and concerns faced by those living with HIV in an African setting. Participants expounded on their perceptions of QoL as depending on environmental conditions (work, finances), perceived usefulness of life (to other people such as family), appreciation of life (contented with oneself), ability to cope with life (good health, stigma, disclosure and social support) and satisfaction with life in general.

Thanks to early diagnosis of HIV, the widespread availability of ART and the prospect of prolonged life, those living with HIV no longer experience the same dramatic life disruption that they once did. Although participants expressed hope for the future, the fundamental challenges associated with living with HIV such as stress, stigma, poverty and depression have not changed over time and continue to have an adverse impact on QoL for many. These factors need to be underscored and addressed when managing these patients.

Although the majority of participants recognised that ART and good health contribute to their QoL, other challenges such as access to medical care, psychological support, social support, in addition to a lack of economic empowerment must be addressed both in a clinical setting and through public health policies.

Happiness and satisfaction with life are fundamental in determining QoL for Ugandan patients living with HIV. While good QoL is an aspiration for those living with HIV in resource-poor settings, for many there remains significant uncertainty as to whether it can be attained.

## Abbreviations

ART: Antiretroviral therapy; ARV: Antiretroviral drug; HRQoL: Health-related quality of life; IDIs: In-depth interviews; MOS-HIV: Medical outcomes study HIV; QoL: Quality of life; UNAIDS: The Joint United Nations Programme on HIV/AIDS; UNAIS: Uganda National AIDS survey; WHO: World Health Organization.

## Competing interests

The authors declare that they have no competing interests.

## Authors’ contributions

JS, FM, AK and DMM participated in the conception and design of the study, DMM collected the data, JS and DMM analysed the data and JS, FM and DMM interpreted the data. DMM drafted the initial manuscript. All authors read and approved the final manuscript, contributed to the critical review and made substantial contributions to its content.

## Pre-publication history

The pre-publication history for this paper can be accessed here:

http://www.biomedcentral.com/1471-2458/14/343/prepub
